# Phototransformation of the Aqueous Fungicide Thiabendazole by Direct Photolysis and Heterogeneous Photocatalysis

**DOI:** 10.1021/acs.iecr.4c04696

**Published:** 2025-06-03

**Authors:** Zenydia Marín, Diana Rojas, Isabel Fernández Pérez, J. Arturo Santaballa, Moisés Canle

**Affiliations:** Departamento de Química, Facultade de Ciencias & CICA, 16737 Universidade da Coruña, A Coruña E-15071, Spain

## Abstract

This study focuses on the degradation of the postharvest fungicide thiabendazole (**TBZ**), which is widely used in citrus production and veterinary medicine. Although **TBZ** is effective in controlling fungal infections, its residues have been detected in agricultural runoff, industrial wastewater, and surface and groundwater, demonstrating its resistance to conventional water treatment methods. Additionally, **TBZ** is highly toxic to aquatic organisms and has been classified as a possible carcinogen at high doses. To address this issue, advanced oxidation processes (AOPs), particularly heterogeneous photocatalysis, have emerged as promising solutions. Titanium dioxide (TiO_2_) is one of the most effective photocatalysts due to its chemical stability, low cost, and strong oxidizing ability when exposed to UV radiation. This study examines the phototransformation of **TBZ** using TiO_2_ P25, evaluating its degradation products and proposing a set of reaction pathways. The phototransformation of aqueous thiabendazole (**TBZ**), using commercial TiO_2_–P25 as a photocatalyst and UV irradiation (mainly 365 nm), is much faster than using UV irradiation (254 nm) alone. All kinetic runs were carried out at natural pH (ca. 6.1) and 298 K. The rate constant for photocatalyzed **TBZ** disappearance was ca. (4.8 ± 0.5)·10^–3^ s^–1^ (*t*_1/2_ ca. 3.5 min), with a mineralization of ca. 67% after 2 h, relative to the initial concentration of fungicide. Aqueous **TBZ** also underwent 254 nm induced phototransformation, with a rate constant of disappearance ca. (5.0 ± 0.1)·10^–5^ s^–1^ (*t*_1/2_ ca. 221 min), and just 3% of mineralization after 340 min. The quantum yield for direct phototransformation was Φ = 0.05, and the reactive species was identified as the singlet excited state of **TBZ**, as the concentration of dissolved O_2_ does not affect the phototransformation process. Up to 11 photoproducts were identified by HPLC-MS, the main one being benzimidazole. A suitable set of reaction pathways that may explain these products are proposed.

## Graphical abstract

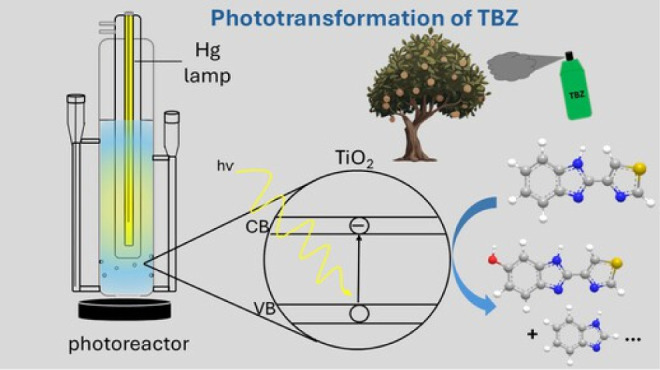

## 1 Introduction

In the past decade, new highly sensitive analytical techniques have emerged, particularly GC/MS,^1^ which have enabled the detection of persistent and emerging organic pollutants (POPs) in the environment, such as pharmaceuticals and personal care products, veterinary drugs, pesticides, endocrine-disrupting compounds (EDCs), hormones, nanomaterials, and fungicides, among others.^2,3^

This study focuses on the degradation of the postharvest fungicide thiabendazole (**TBZ**, Figure 1), which is widely used in citrus production to enhance fruit quality and yield. **TBZ** belongs to the noncarbamate benzimidazole subgroup.^4^ Its use is authorized by the European Union and other regulatory agencies^5–7^ for controlling diseases in fruits and vegetables, including mold, rot, blight, and spotting caused by pathogens such as Pseudomonas fluorescens *, Pseudomonas sp.,* and Badhamia utricularis.^7,8^ In citrus fruits, **TBZ** prevents infections caused by Penicillium digitatum, Penicillium italicum *, Diplodia sp., Phomopsis sp.,* and other postharvest pathogens.

**Figure 1:**
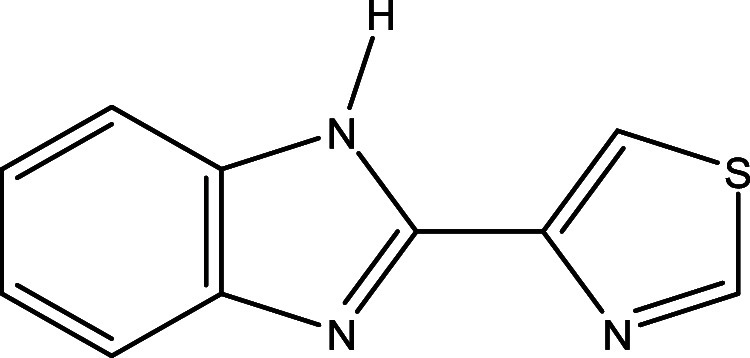
Chemical structure of thiabendazole (TBZ).

Beyond its agricultural applications, TBZ is also used in veterinary medicine to treat hepatic diseases in sheep and goats. Despite its benefits, only a small fraction of **TBZ** effectively fulfills its intended purpose, while the remainder contaminates the environment, primarily through soil and water. **TBZ** can also generate numerous metabolites, many of which are highly soluble in water and pose potential risks to living organisms.^9^ Postharvest fungicides are typically removed through washing, and the resulting residues are directly discharged into sewage systems. **TBZ** residues have been detected in agricultural runoff as well as in industrial and urban wastewater.^10–14^ Furthermore, **TBZ** has been found in surface, groundwater, and wastewater at concentrations of up to 3.3 mg·L^–1^,^10,15–17^ demonstrating its resistance to conventional wastewater treatments.^17–19^

A study conducted by this research group found **TBZ** concentrations of 70 g/day at the inflow of the wastewater treatment plant (WWTP) in the city of A Coruña (NW Spain). This is particularly worrying because **TBZ** is highly toxic to fish and freshwater and estuarine invertebrates. In humans, **TBZ** generally exhibits low acute toxicity (class III).^20^ However, U.S. EPA has classified it as a possible carcinogen at sufficiently high doses capable of disrupting thyroid hormone balance.^7^ Studies in mice have shown that high doses of **TBZ** may affect the urinary tract and the reproductive system in both sexes.^21,22^

It therefore becomes urgent to develop alternative methods for the elimination or reduction of persistent organic pollutants, such as **TBZ** and its degradation products. Among the most promising technologies for treating emerging contaminants in wastewater are advanced oxidation processes (AOPs), which generate hydroxyl radicals (HO^•^). Heterogeneous photocatalysis has shown remarkable efficiency in the degradation of fungicides.^23,24^

In particular, heterogeneous photocatalysis with TiO_2_ has gained significant attention, as this material is one of the most widely used photocatalysts due to its chemical stability, relatively low toxicity, affordability, and ease of synthesis. Additionally, when exposed to UV or near-UV radiation, it becomes a powerful oxidant.^18,23–26^ The absorption of photons with an energy exceeding 3.2 eV generates a highly reactive surface with excited electrons and holes, which are responsible for the oxidation of organic contaminants through the photocatalytic process.

The three most studied TiO_2_ polymorphs found in nature are anatase, rutile, and brookite. In photocatalytic applications, the most commonly used commercial TiO_2_ is AEROXIDE TiO_2_ P25 (Evonik Degussa Corporation), which consists of a mixture of anatase and rutile crystalline phases in a 4:1 mass ratio, along with a specific amount of amorphous TiO_2_.^27^

Other AOPs, such as the photo-Fenton process and UV/H_2_O_2_, have also proven to be highly effective in the degradation of fungicides,^17,28,29^ though not as much as heterogeneous photocatalysis.

This study focuses on the phototransformation of **TBZ** by using heterogeneous photocatalysis with TiO_2_ P25. Additionally, its degradation products are analyzed, and a set of reaction pathways is proposed.

## 2 Experimental Section

### 2.1 Reagents and Chemicals

4-(1*H*-benzo­[*d*]­imidazol-2-yl)­thiazole Pestanal (**TBZ**), an analytical standard, was purchased from Riedel-de-Haën. A standard of the compound (purity >98.0%) used for byproduct identification was obtained from the same company. All products were used without further purification. Commercial TiO_2_ P25 powder from Evonik was obtained from Degussa Portuguesa. The solvents used for HPLC analysis were methanol, HPLC grade from J.T. Baker, and ultrapure water, produced *in situ* with a Direct-Q Millipore system. Double distilled water (in which organic matter had previously been oxidized by boiling with KMnO_4_) was used for the **TBZ** solutions.

### 2.2 Direct Phototransformation Experiments: Steady-State Irradiation

Experiments were performed in a 750 mL glass immersion photochemical reactor charged with 500 mL of **TBZ** in aqueous solution. The reactor was equipped with a UV lamp, located axially and held in a quartz immersion tube, and a circulating water quartz jacket was used to keep the temperature of the solution within 298.0 ± 0.1 K. The radiation source was a Heraeus TNN 15/32 low-pressure Hg vapor lamp (3 W nominal radiant flux) with a main emission line at 254 nm. The photon flux inside the photoreactor, measured by potassium ferrioxalate actinometry,^30^ was 3.33·10^–8^ Einstein·s^–1^. In a typical experiment, the initial concentration of **TBZ** was set at 0.05 mM. Before inserting the lamp, previously ignited and warmed up for 15 min out of the reaction medium, the solutions were gas-saturated (Ar, air, or O_2_ as required) and magnetically stirred for 30 min. Then, the solutions were irradiated with UV light at a constant stirring speed. Reactions were stopped after 320 min of irradiation.

### 2.3 Photocatalytic Degradation Experiments

Irradiation experiments were carried out with a 250 mL Heraeus UV reactor system open to the air. The light source was a Heraeus TQ 150 medium-pressure Hg vapor lamp with main emission lines at 254, 313, 365, 436, 546, and 578 nm. A DURAN 50 glass jacket allows cooling by water circulation to keep a constant temperature and acts also as an optical cutoff of UVB and UVC radiation. The resulting main emission line was at 365 nm and the photon flux was 2.38·10^–6^ Einstein·s^–1^, as determined by potassium ferrioxalate actinometry.^30^

In a typical experiment, the initial concentration of **TBZ** was set at 0.05 mM. The amount of suspended TiO_2_ was kept at 0.5 g·L^–1^. A dark experiment was carried out for 180 min, checking that the adsorption–desorption equilibrium was established within 60 min without significant spectral changes after this time. Thereafter, the lamp, previously ignited and warmed, was inserted into the reaction medium. The solutions were stirred at a constant stirring speed. Reactions were stopped after 120 min of irradiation. Aliquots were withdrawn from the reactor at different reaction times and centrifuged for 15 min at 17,746 g to separate the photocatalyst immediately before analysis.

### 2.4 Analytical Procedures

The phototransformation of **TBZ** was monitored by UV–vis spectrophotometry with a Biochrom Libra S270 double-beam array spectrophotometer, using standard quartz cells with a 10 mm path length and 3.5 mL capacity. Monitoring was also carried out by HPLC-PAD, with a Spectra System of Thermo Fisher Sci., instrument equipped with a photodiode array detector (UV 6000 LP), a C18 Hypersil ODS-2 column (150 mm × 4.6 mm, 5 μm particles), and a pump (P4000). The injection was 50 μL, and the flow rate was 0.7 mL·min^–1^. An isocratic mobile phase composition was used: methanol-aqueous ammonium acetate 20 mM pH 6.8 (65:35).

Photoproducts identification was carried out by comparison with authentic samples, when available. Furthermore, an HPLC-MS system with a Thermo LTQ Orbitrap Discovery mass spectrometer was used, with electrospray ionization in positive mode (ESI^+^), coupled to a U-HPLC Accela system.

Total organic carbon (TOC) was measured by using a Shimadzu TOC-V CSN analyzer.

## 3 Results and Discussion

### 3.1 Direct Phototransformation

The UV–vis spectrum of an aqueous solution of **TBZ** shows two absorption bands (Figure 2, spectrum at *t* = 0 min); the more intense, at 298 nm, with ε = 25,000 ± 730 M^–1^·cm^–1^, can be attributed to a *n* → π* transition, and the less intense at 240 nm, with ε = 15,756 ± 422 M^–1^·cm^–1^, assigned to a π → π* transition. The inset in Figure 2 shows the Lambert–Beer law for **TBZ** at 298 nm. These bands do not show any alteration with the pH of the medium.

**Figure 2:**
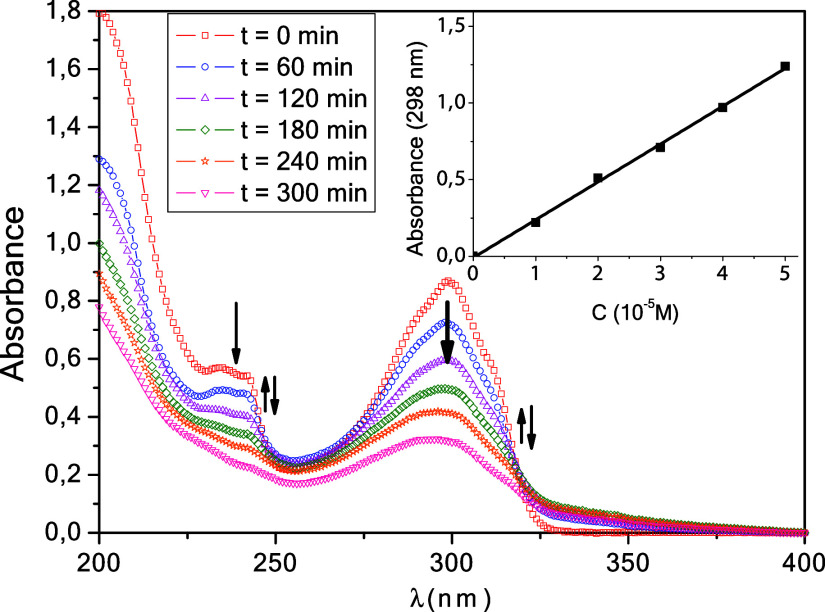
Time-resolved UV–vis spectrum of an aqueous solution of **TBZ** upon irradiation at λ_exc_ = 254 nm. [TBZ]_0_ ≈ 0.05 mM, pH_nat_ ≈ 6.12; *P*(O_2_) = 21% (v/v), *T* = 298.0 K. Inset: **TBZ** Lambert–Beer law at 298 nm.

Figure 2 also shows the time-resolved UV–vis spectra of an aerated aqueous solution of **TBZ** (0.05 mM) upon irradiation at 254 nm at pH 6.12, a natural pH (pH_nat_) of the **TBZ** solution. Under these conditions and considering that p*K*_a_ (**TBZ**) ≈ 4.64,^31^ almost 3% of **TBZ** is in its protonated form. Monitoring of the reaction was also carried out by HPLC-PDA for 320 min. The percentage of phototransformation of **TBZ** was 72%. However, the TOC results show that only 3% of the initial amount of fungicide was mineralized in this period.

As the reaction progresses, there is a continuous decrease in absorbance at 240 and 298 nm, with an increase at 250 and 325 nm, evidencing the formation of photoproducts, which also undergo phototransformation over time. During the first 180 min, an isosbestic point is observed at 319 nm, indicating a single clean process. After 120 min, the maximum wavelength starts to shift toward lower energies due to the absorbance of the different appearing species. Consequently, after 180 min, an absorbance increase at 250 and 325 nm is observed, followed by a subsequent decrease. When treating these processes as consecutive first-order reactions, errors of the same magnitude as the rate constants or even larger were obtained. For this reason, it was decided to restrict the fit to the first process, using for this a first-order kinetic model (Figure 3 and Table 1).

**Figure 3:**
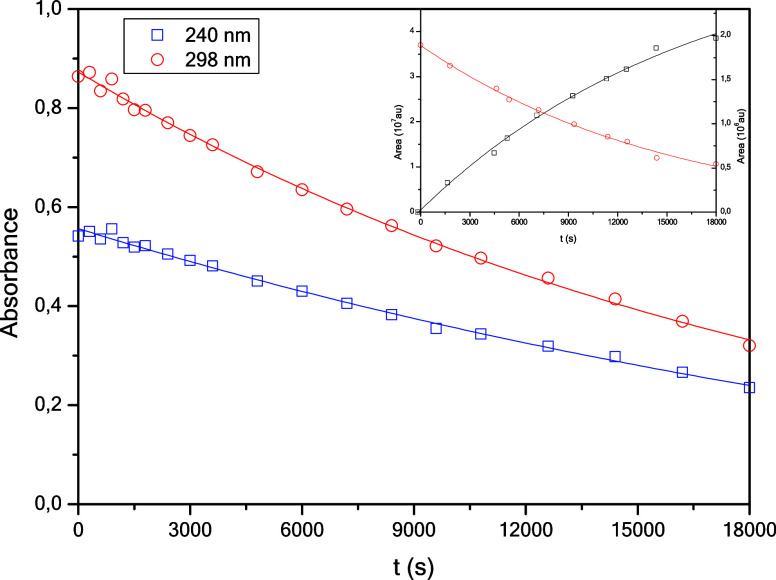
Absorbance vs time profiles for the direct phototransformation of **TBZ** in aqueous solution. λ_exc_ = 254 nm, [TBZ]_0_ ≈ 0.05 mM, pH_nat_ ≈ 6.12; *P*(O_2_) = 21% (v/v), *T* = 298.0 K. Inset: monitoring was performed by HPLC-PDA, λ_detection_ = 298 nm.

**Table 1:** Rate Constants Obtained for the Direct Phototransformation of **TBZ** in Aqueous Solution^a^

λ/nm	(*k*_obs_± σ_k_)·10^–5^/s^–1^
240	3.1 ± 0.4
298	4.3 ± 0.1

a λ_exc_ = 254 Nm, [TBZ]_0_ ≈ 0.05 mM, pH_nat_ ≈ 6.12; *P*(O_2_) = 21% (V/V), *T* = 298.0 K

The inset in Figure 3 shows the result of monitoring the reaction by HPLC-PDA (detection at 298 nm), in which the disappearance of **TBZ** (*k* = (6 ± 1)·10^–5^ s^–1^), accompanied by the appearance of a photoproduct (*k* = (6 ± 1)·10^–5^ s^–1^) is observed. The agreement between the rate constants indicates that they correspond to the same process.

#### 3.1.1 Effect of Dissolved O_2_

This study was carried out by irradiating at 254 nm a 0.05 mM aqueous **TBZ** solution, bubbling with O_2_ or Ar (100% or 0% O_2_). The gas supply was kept during the experiments.

The presence of O_2_ in the reaction medium did not affect the direct phototransformation of **TBZ**, since the observed rate constant was ca. (5.0 ± 0.1)·10^–5^ s^–1^ with all three concentrations studied (0, 21, and 100% of O_2_), which suggests that just ^1^ **TBZ** is involved in the phototransformation and not ^3^ **TBZ**.

The **TBZ** phototransformation quantum yield was calculated from the following equation:^26,32–34^

$$\phi_{\mathrm{phototransformation}}=\frac{k_{obs}}{2.303\cdot I_{\lambda }\cdot \epsilon_{\lambda }\cdot l}$$

where Φ_phototransformation_ is the phototransformation quantum yield, *k*_obs_ (s^–1^) is the phototransformation observed first-order rate constant, *I*_λ_ (Einstein L^–1^·s^–1^) is the light intensity at wavelength λ, and ϵ_λ_ (cm^–1^·mol·dm^–3^) is the molar absorptivity at wavelength λ and is the cell path length (cm). The obtained photodegradation quantum yield (Φ_phototransformation_) for **TBZ** was 0.05, indicating that, besides phototransformation, ^1^ **TBZ** also deactivates through photophysical processes.

### 3.2 Photocatalytic Degradation

Commercial TiO_2_ P25 was used as a photocatalyst in the catalyzed TBZ phototransformation. The catalyst load was 0.5 g·L^–1^. After the equilibration in the dark, where 4.4% of **TBZ** was adsorbed onto the catalyst, the photocatalytic degradation of TBZ was performed at the natural pH of the mixture. **TBZ** underwent a complete phototransformation in less than 15 min. TOC results showed that 67% of the fungicide mineralized after 120 min.

Figure 4 shows the simultaneous disappearance of **TBZ** and the appearance of four photoproducts in the first 10 min, which subsequently begin to photodegrade. **TBZ** decays following first-order kinetics, with a rate constant *k* = (5.0 ± 0.5)·10^–3^ s^–1^. Four different intermediates are observed at 3.2, 4.8, 5.4, and 6.4 min retention times. In a consecutive process, each one reaches its maximum concentration after ca. 10 min and then disappears, each with a different rate. No fits are provided for these consecutive processes. Most of the TOC remaining at 120 min, 33%, correspond to the intermediate with HPLC 3.2 min retention time.

**Figure 4:**
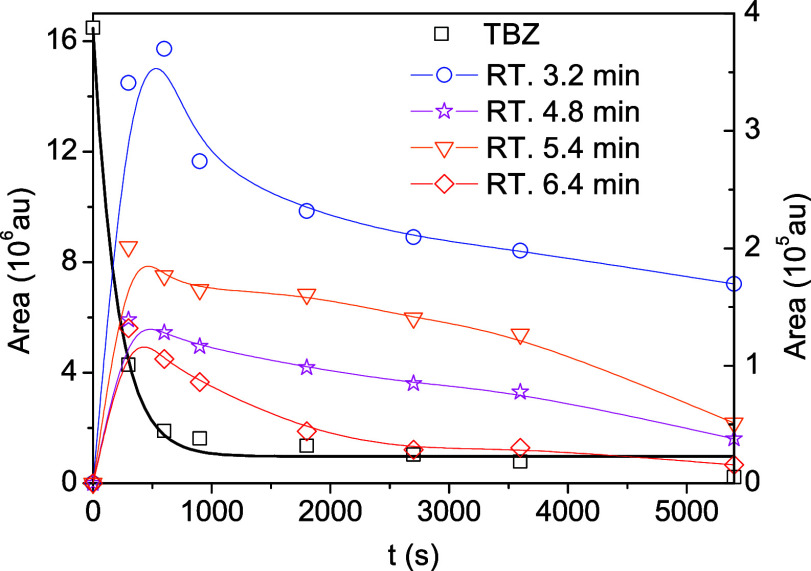
Area vs time profiles for the photocatalyzed transformation of **TBZ** in aqueous solution. λ_exc_ = 365 nm, [TBZ]_0_ ≈ 0.05 mM, [TiO_2_–P25] = 0.5 g·L^–1^, pH_nat_ ≈ 6.12, *P*(O_2_) = 21% (v/v), *T* = 298.0 K.

### 3.3 Identification of Photoproducts by HPLC-MS

The same photoproducts were identified by HPLC-MS for both direct phototransformation, after 340 min, and photocatalytic transformation, after 120 min, of **TBZ** (0.05 mM) aerated at pH_nat_ 6.12 (Table 2). The observed *m*/*z* ratios were coincident in all cases, with the corresponding calculated *m*/*z* ratios to within 0.0001.

**Table 2:**
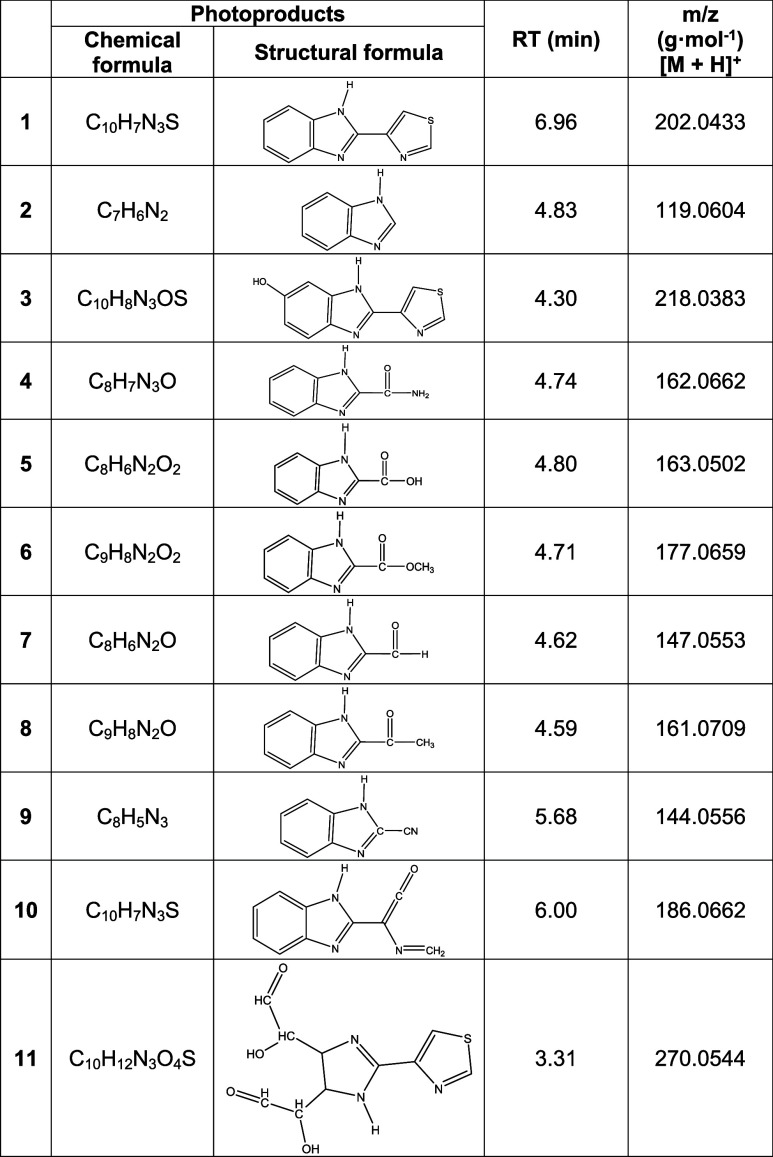
Observed Products, by HPLC-MS, of Direct and Catalyzed Phototransformation of **TBZ** in Aqueous Solution after Reaction for 120 min of Reaction^a^

a [TBZ]_0_ ≈ 0.05 mM, [TiO_2_–P25] = 0.5 g·L^–1^, pH_nat_ ≈ 6.12, *P*(O_2_) = 21% (V/V), *T* = 298.0 K.

Products **2** and **4** were also identified by Jacob et al.^35^ while studying the degradation of **TBZ** supported on glass upon sunlight irradiation of thin films, and of **TBZ** in 20% MeOH solution, in this last case using UV light. The same two photoproducts were identified later by Watkins,^36^ who irradiated **TBZ** in MeOH with a medium-pressure Hg lamp. Mahran et al.^37^ found photoproducts **2**, **4,** and **6** in the photooxidation of **TBZ** in 1% MeOH, irradiating with λ > 313 nm, in the presence and absence of methylene blue. Murthy et al.^38^ identified photoproducts **2**, **4**, **7**, **8,** and **9** upon photolysis of aqueous **TBZ** in the presence of humic and fulvic acids, irradiating at λ ≥ 290 nm.

Calza et al.^12^ carried out the heterogeneous photocatalysis of aqueous 7.5 mM **TBZ** at 50 °C using a 1500 W Xe lamp, cutting off wavelengths below 340 nm, and filtering the solutions with 0.45 μm filters (material not specified); they identified photoproducts **2**, **3**, **11**.

## 4 Reaction Pathways

Based on the kinetic results and the identified photoproducts, a detailed set of photoconversion pathways has been proposed to account for the observed photoproducts (Figure 5). **TBZ** absorbs a photon and is excited into the singlet state ^1^ **TBZ**, which, via different homolysis and/or heterolysis processes, gives rise to different photoproducts (**2–11**), with the participation of water molecules present in the reaction medium.

**Figure 5:**
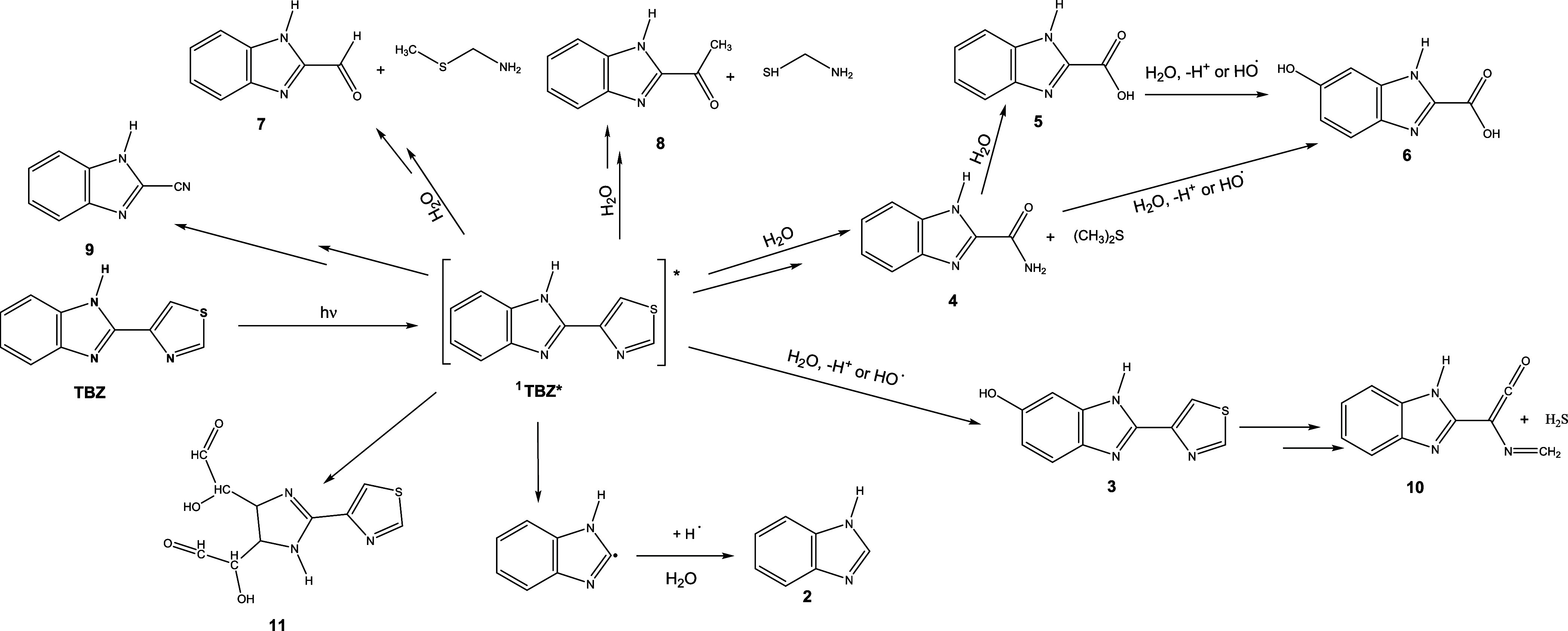
Reaction pathways for the direct phototransformation and heterogeneous photocatalysis of **TBZ** in aqueous solution.

The breaking of homolytic bonds of ^1^ **TBZ**, which is followed by hydrolysis and the addition of H**^•^** that gives way to the formation of **2** (1*H*-benzo­[*d*]­imidazole), which is the main photoproduct. ^1^ **TBZ** by hydrolysis or by the addition of HO^•^ and deprotonation gives rise to the product **3.** The byproduct **3** after a ring opening by C–S scission and subsequent oxidation yields **10** and hydrogen sulfide molecule. Hydrolysis of ^1^ **TBZ** could lead to **4**, **7**, **8**, with the formation of dimethyl sulfide, (methylthio)­methanamine, and aminomethanethiol, respectively. Photoproduct **4** undergoes hydrolysis and gives rise to product **5**, which by hydrolysis or by the addition of HO^•^ and the H^+^ loss leads to **6**, which could also be obtained by a route like the previous one from photoproduct **4**. ^1^ **TBZ** after undergoing successive hydroxylations gives rise to photoproduct **11**. Finally, photoproduct **9** is obtained from the ring opening by C–S scission.

## 5 Conclusions

Up to 72% of **TBZ** was phototransformed under 254 nm UVA radiation after 340 min of reaction in aqueous solution at natural pH (ca. 6.1) and 298 K; however, mineralization percentages, determined as TOC, decreased to 3%. The phototransformation quantum yield was ca. 5%. The amount of dissolved oxygen did not affect the process.

In the photocatalytic regime, using TiO_2_ P25 powder as the photocatalyst, the **TBZ** phototransformation yield was ca. 100% in less than 15 min under UVC and Vis radiation (the main excitation wavelength being 365 nm, aqueous solution at natural pH and 298 K); this time the corresponding percentage of mineralization was 67% after 2 h.

Both processes were monitored by both UV–vis spectrophotometry and HPLC-PDA. As stated before, the observed photocatalytic process was much faster (*t*_1/2_ = 2.3 min) than direct photolysis (*t*_1/2_ = 221 min). Eleven photoproducts have been identified using HPLC-MS after 120 and 340 min reaction, photocatalyzed and direct phototransformation, respectively; the main one being benzimidazole. A suitable set of phototransformation pathways has been proposed for all of them; in general, the observed photoproducts have a lower molecular mass and higher polarity than thiabendazole.
